# Incremental cost-effectiveness pharmacoeconomic assessment of hepatitis C virus therapy: an approach for less wealthy members of the common market

**DOI:** 10.3325/cmj.2016.57.582

**Published:** 2016-12

**Authors:** Diana Mance, Davor Mance, Dinko Vitezić

**Affiliations:** 1 Department of Physics, University of Rijeka, Rijeka, Croatia; 2 Faculty of Economics, University of Rijeka, Rijeka, Croatia; 3 School of Medicine, University of Rijeka, Rijeka, Croatia; 4 University Hospital Centre Rijeka, Rijeka, Croatia

## Abstract

**Aim:**

To develop a new method of health-economic analysis based on a marginal approach.

**Methods:**

We tested the research hypothesis that a detailed comparative *a priori* incremental cost-effectiveness analysis provides the necessary input for budget impact analysis about the proper order of introduction of new therapies, and thus maximizes the cost-effectiveness bounded by the total budget constraint. For the analysis we chose a combination therapy for the treatment of hepatitis C virus (HCV) genotype 1 (GT1) infection, which was approved by the European Medicine Agency in 2015. We used the incremental cost-effective approach to assess the increase in the percentage of patients achieving sustained virological response (SVR) and the expenditure per additional SVR modulated by the new therapy's market entrance dynamics. Patient subpopulations were differentiated by their response to previous treatment, presence of cirrhosis, and HCV GT1 subtype. Final parameters were estimated by Monte Carlo simulations.

**Results:**

The new combination therapy had high efficacy, shorter duration, and was better tolerated than alternative interventions. The research hypothesis was confirmed: gradual introduction of the new therapy on the market, based on *a priori* incremental cost-effectiveness analysis, would result in average increase in successfully treated patients by 20%-40%, while additional costs would approximately be between 8%-40%, ie, €21,000-52 000 per additional patient achieving SVR.

**Conclusion:**

We showed the new combination therapy to be cost-effective for certain patient subpopulations, especially for experienced cirrhotic HCV GT1 patients. Results of the analysis are in agreement with the latest recommendations for HCV patients’ treatment in Croatia. This economic evaluation could serve as a starting point for negotiations between pharmaceutical industry and insurance companies.

For European Union (EU) Member States (MSs), being part of the common market means that prices of drugs/therapies cannot be adjusted to individual markets’ optima. Namely, the price is determined by the EU dominant market, formed by Germany, France, United Kingdom, Italy, and Spain (EU5). The EU Treaty specifically forbids price discrimination by direct segmentation, ie, discrimination according to the country of residence or nationality (1). As a consequence, patients from poorer EU member states are either paying higher prices or are left without adequate health care because of its unaffordability.

Another challenge for those dealing with health-economic aspects of the introduction of a new medicine on the low/middle income EU MSs’ market, is imposed by Health Management Organizations (HMOs) that do not use health state or utility based allocation mechanisms, such as quality adjusted life years (QALYs), health adjusted life years (HALYs), or disability adjusted life years (DALYs), but base their decisions on ordinary budget impact analyses (BIA). New, highly effective therapies can be extremely expensive, and if the decision about their inclusion on the reimbursement list is based only on ordinary BIA, the impact on low/middle income EU MSs’ HMOs' budgets might be unacceptable.

The aim of the article is to develop a new method of health-economic analysis based on a marginal approach. Using this approach it is possible to show that, despite the high price, the new medicine/therapy can still be acceptable even for less wealthy EU MSs. In order to achieve the aim, we tested the research hypothesis that a detailed comparative *a priori* incremental cost-effectiveness analysis provides the necessary input for BIA about the proper order of introduction of new therapies, and thus maximizes the cost-effectiveness bounded by the total budget constraint.

## Data and methods

### An example of extremely expensive therapy: new hepatitis C virus combination therapy

As an example of a new and expensive therapy we chose the ombitasvir, paritaprevir, ritonavir, and dasabuvir (OBV/PTV/r/DSV) combination therapy for hepatitis C virus (HCV) infection. OBV/PTV/r/DSV combination therapy is an all-oral, interferon-free therapy that suppresses the viral replication in infected cells, leading to virus elimination (2). The therapy combines direct acting antivirals (DAAs) with different mechanisms of action and resistance profiles that do not overlap, targeting the HCV in its different life cycle stages (2).

From pharmacological point of view, OBV/PTV/r/DSV therapy meets the requirements for new therapeutic protocols: better efficacy, shorter duration, and better tolerance in comparison to currently available therapies (2,3). It is, along simeprevir- (SIM) and sofosbuvir- (SOF) based therapies, a new HCV therapy that has been approved in Europe during the last three years. Due to their sky-high prices, these therapies are often referred to as “1000 $ per pill” therapies. Their prices breach the budget constraints of many countries and impose additional allocation requirements on decision-makers (4-7).

### An example of low/middle income European Union Member State: Croatia

Croatia was chosen as an example of low/middle income EU MS. Croatian Health Insurance Fund (CHIF), the national and only social health provider in Croatia, does not use health state or utility based allocation mechanisms (8), such as QALYs, HALYs, or DALYs. Instead of this, the CHIF’s decisions are based on BIA. Croatia is a low HCV prevalence country (<2% chronically HCV infected individuals), with genotype 1 (GT1) as the most common genotype (approximately 60% of HCV population) (7,9-11). The predominant HCV GT1 subtype is 1b (64%), followed by subtype 1a (22%) and unknown subtype infection or mixed infection (14%) (10). Due to the lack of official epidemiological data, the number of experienced patients eligible for new treatment (90-100 per year) was estimated according to the opinion of Croatian practitioners dealing with HCV patients. It is important to emphasize that this range is a rough estimate and should not be used for other purposes.

### Health-economic analysis

The analysis was performed from the CHIF’s perspective and only the short-term outcomes of the therapy (one year) were analyzed. Since OBV/PTV/r/DSV therapy lasts 12-24 weeks and has resulted in high sustained virological responses (SVR) in clinical trials (2), we considered the short term therapy outcomes to be more relevant for the health-economic analysis than the life-time horizon. The analysis was performed at the time when OBV/PTV/r/DSV was about to enter the Croatian market (year 2015). The therapies present on the market at that time were pegylated interferon (pegIFN), boceprevir (BOC+pegIFN), telaprevir (TPV+pegIFN), and simeprevir (SIM+pegIFN)-based regimens. These therapies were used as alternative therapies to OBV/PTV/r/DSV in the study. Therapies’ costs were limited to direct costs and calculated according to publicly available CHIF’s medication prices (12) and currently valid therapy protocols for HCV GT1 patients (13). Therapy prices were expressed in € 2015 (14). Ribavirin (RBV) is donated to the CHIF by pharmaceutical companies so its price was not used in calculations. For this reason, although they include RBV the therapies are designated as pegIFN, BOC+pegIFN, TPV+pegIFN, SIM+pegIFN, and OBV/PTV/r/DSV. In calculations, pegIFN alfa-2a price was used, since experience from practice suggests that this pegIFN type has been mostly used in Croatia (15).

The used inputs also include duration and SVR rates for OBV/PTV/r/DSV therapy and alternative regimens (Table 1). These were derived from clinical trials and published literature for OBV/PTV/r/DSV (2), pegIFN (13,16,17), TPV (13,16,18,24), BOC (13,18-23), and SIM-based regimens (19,25). SVR rates were expressed as ranges of values found in the cited literature.

**Table 1:** SVR ranges and therapy durations for different HCV GT1 patient subgroups and treatment regimens*

	OBV/PTV/r/DSV				pegIFN		BOC+pegIFN		TPV+pegIFN		SIM+pegIFN	
HCV GT1 patient subgroup	no cirrhosis		cirrhosis		no cirrhosis	cirrhosis	no cirrhosis	cirrhosis	no cirrhosis	cirrhosis	no cirrhosis	cirrhosis
SVR (%)												
naive		1a: 95.9 1b: 100	1a: 94.6 1b: 100		35-50	33-38	52-67	52-55	64-85	53-71	68-84	57-60
relapsers		1a: 94 1b:100	1a, 1b: 100		13-35	7-25	75-93	34-54	83-88	74.2-84	57-82	46-74
partial responders		1a, 1b: 100	1a: 100 1b: 85.7		0-43	5-20	52-67	34-38	54-79	34-40	57-79	46-82
null responders		1a: 95.4 1b: 100	1a: 92.9 1b: 100		0-8	0-10	38-39	0-34	33-43	14-19	33-66	31-46
Therapy duration (weeks)												
naive		12		1a: 24 1b: 12	24/48	48	28/48	48	24/48	48	24	24
relapsers		12		1a: 24 1b: 12	48	48	48	48	24/48	48	24	24
partial responders		12		1a: 24 1b: 12	48	48	48	48	24/48	48	48	48
null responders		12		1a: 24 1b: 12	48	48	48	48	48	48	48	48

*SVR – sustained virological response; HCV GT1 – hepatitis C virus genotype 1; 1a, 1b – HCV GT1 subtypes; OBV/PTV/r/DSV – ombitasvir, paritaprevir, ritonavir and dasabuvir (2); pegIFN – pegylated interferon (13,16,17); BOC – boceprevir (13,18-23); TPV – telaprevir (13,16,18,24); SIM – simeprevir (19,25).

Comparisons of health-economic outcomes were made across treatment regimens for 4 groups of naive patients and 12 groups of experienced HVC GT1 patients. Grouping was made according to genotype (GT1a and GT1b, where GT1a encompassed mixed type as well), response to prior treatment (relapse, partial response, null response), and presence of cirrhosis. The analyzed health-economic outcomes were: I) cost-effectiveness; II) budget impact; III) number of successfully treated patients; IV) expenditures per successfully treated patient; and V) incremental cost-effectiveness ratio (ICER).

The specificity of this study is that the cost-effectiveness of the new combination therapy was the basis for other analyzed health-economic outcomes. Estimated savings per SVR brought by OBV/PTV/r/DSV determined the scenarios and the dynamics of taking over the market shares. We simulated that OBV/PTV/r/DSV therapy gradually took over the market shares starting with patient subgroups with the greatest projected savings per SVR toward subgroups with lower projected savings.

For HCV GT1 experienced patients, dual pegIFN+RBV therapy has very low SVR rates: patients with partial and null response have SVR rates as low as 0% (Table 1) (16). Due to this and the fact that dual therapy has not been included in CHIF’s guidelines for treatment of HCV GT1 experienced patients (12), the calculation for experienced patients considering dual therapy was not conducted. BOC, TPV, and pegIFN, therapies gain more success in the treatment of non-GT1 HCV patients than of GT1 patients (26). Therefore, since GT 2, 3, 4, 5, and 6 can be successfully treated with therapies that are significantly cheaper than OBV/PTV/r/DSV, these genotypes were not included in the present analysis.

### Statistical analysis

The results of cost-effectiveness analyses and the number of patients and SVR rates for specific regimens and patient groups were varied across complete ranges in 10 000 Monte Carlo simulation runs. Calculations were performed in Microsoft Excel (Microsoft Corp., Redmond, WA, USA). Monte Carlo simulation results were analyzed by ANOVA and Tukey post-hoc test. Differences were considered statistically significant at the level of *P* < 0.05. Statistical analysis was performed in Statistica 12.0 software (StatSoft Inc, Tulsa, OK, USA).

## Results

### Cost-effectiveness

**Figure 1:**
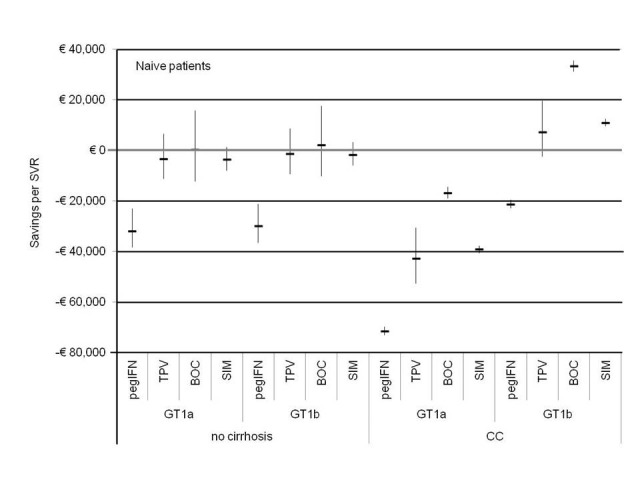
Results of Monte Carlo simulation: average savings per SVR (horizontal lines) and savings’ ranges (vertical lines) of OBV/PTV/R/DSV therapy compared to rival therapies for HCV GT1 naive patients with and without cirrhosis. Positive values: costs of OBV/PTV/r/DSV therapy per SVR are expected to be lower than costs of rival therapies. Negative values: costs of OBV/PTV/r/DSV therapy per SVR are expected to be higher than costs of rival therapies. SVR – sustained virological response, HCV GT1 – hepatitis C virus genotype 1, 1a, 1b – HCV GT1 subtypes, OBV/PTV/r/DSV – ombitasvir, paritaprevir, ritonavir, and dasabuvir, pegIFN – pegylated interferon, TPV – telaprevir + pegIFN, BOC – boceprevir + pegIFN, SIM – simeprevir + pegIFN, CC – compensated cirrhosis.

**Figure 2:**
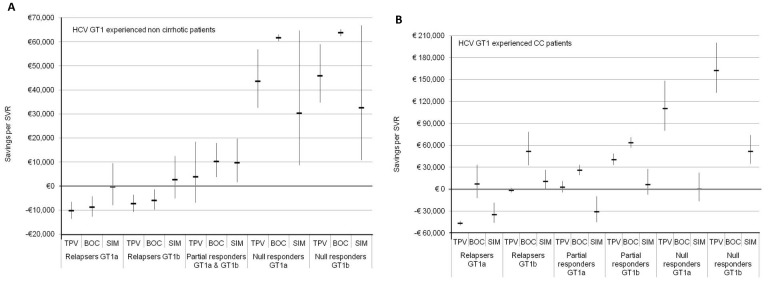
Results of Monte Carlo simulation: average savings per SVR (horizontal lines) and savings’ ranges (vertical lines) of OBV/PTV/r/DSV therapy compared to rival therapies for HCV GT1 (**A**) experienced non cirrhotic patients; (**B**) experienced cirrhotic patients. Positive values: costs of OBV/PTV/r/DSV therapy per SVR are expected to be lower than costs of rival therapies. Negative values: costs of OBV/PTV/r/DSV therapy per SVR are expected to be higher than costs of rival therapies. SVR – sustained virological response, HCV GT1 – hepatitis C virus genotype 1, 1a, 1b – HCV GT1 subtypes, OBV/PTV/r/DSV – ombitasvir, paritaprevir, ritonavir, and dasabuvir, pegIFN – pegylated interferon; TPV– telaprevir + pegIFN; BOC – boceprevir + pegIFN; SIM– simeprevir + pegIFN; CC – compensated cirrhosis.

The results of the cost-effectiveness analysis are presented as average therapies’ costs per SVR (Table 2) and as savings per SVR of OBV/PTV/r/DSV combination therapy in comparison to alternative therapies. Differences between average OBV/PTV/r/DSV costs per SVR and average costs per SVR of alternative regimens were significant (*P* < 0.001). For naive patients the savings are presented in Figure 1, and for experienced patients without cirrhosis and with cirrhosis, respectively, in Figures 2A and 2B. Positive values were obtained when OBV/PTV/r/DSV therapy was projected to be cheaper per SVR than rival therapies (savings), and negative values were obtained when OBV/PTV/r/DSV therapy was projected to be more expensive than rival therapies (expenditures). The figures show arithmetic means and ranges rather than standard deviations in order to cover all possible situations, ie, to show minimum and maximum possible savings/expenditures.

**Table 2:** Direct therapy costs and average therapy costs per SVR (obtained by Monte Carlo simulation) for different HCV GT1 patient subgroups and treatment regimens*

	OBV/PTV/r/DSV		pegIFN		BOC+pegIFN		TPV+pegIFN		SIM+pegIFN		
HCV GT1 patient subgroup	no cirrhosis	cirrhosis	no cirrhosis	cirrhosis	no cirrhosis	cirrhosis	no cirrhosis	cirrhosis	no cirrhosis		cirrhosis
Therapy costs (VAT included) (€)											
naive	45,000	1a: 90 000 1b: 45 000	4200/8400	8400	23 200/32 700	41 900	30 200/34 300	34 300	32 800	32 800	
relapsers	45 000	1a: 90 000 1b: 45 000	8400	8400	32 700	41 900	30 200/34 300	34 300	32 800	32 800	
partial responders	45 000	1a: 90 000 1b: 45 000	8400	8400	32 700	41 900	30 200/34 300	34 300	36 900	36 900	
null responders	45 000	1a: 90 000 1b: 45 000	8400	8400	41 900	41 900	34 300	34 300	36 900	36 900	
Average therapy costs per successfully treated patient (€)											
naive	1a: 46 900 1b: 45 000	1a: 95 200 1b:45 000	15 000	23 700	47 300	78 300	43 600	52 400	43 200	56 000	
												relapsers	1a: 47 900 1b: 45 000	1a: 90 000 1b: 45 000	-	-	39 200	96 900	37 700	43 500	47 600	55 700	
partial responders	45 000	1a: 90 000 1b: 52 500	-	-	55 300	116 000	49 200	93 000	54 700	59 300	
null responders	1a: 47 200 1b: 45 000	1a: 96 900 1b: 45 000	-	-	108 800	-	90 900	207 400	77 400	97 100	

*SVR – sustained virological response; HCV GT1 – hepatitis C virus genotype 1; 1a, 1b – HCV GT1 subtypes; OBV/PTV/r/DSV – ombitasvir, paritaprevir, ritonavir and dasabuvir; pegIFN – pegylated interferon; BOC – boceprevir; TPV – telaprevir; SIM – simeprevir.

In naive patient subgroups, the model projected the standard dual therapy to be the cheapest one, not only when real costs were taken into account but also when costs per achieved SVR were compared (Table 2, Figure 1). Only in the HCV GT1b (CC) subgroup of naive patients, considerable savings per SVR of the OBV/PTV/r/DSV therapy were predicted in comparison to rival protease inhibitor (PI) regimens (Figure 1). In non-cirrhotic null-responders, the introduction of OBV/PTV/r/DSV would result in considerable savings per achieved SVR (Table 2, Figure 2A). Also, the savings per SVR would be realized for non-cirrhotic partial responders who would otherwise be treated with BOC or SIM. For other subgroups of experienced non-cirrhotic patients, OBV/PTV/r/DSV costs were comparable or higher than the costs of rival therapies. In experienced CC patients, OBV/PTV/r/DSV therapy was, in most of the cases, the cheapest. The only cheaper options in experienced CC population were SIM based therapy for GT1a partial responders and both SIM and TPV regimens for GT1a relapsers (Table 2, Figure 2B).

### Budget impact analysis

**Figure 3:**
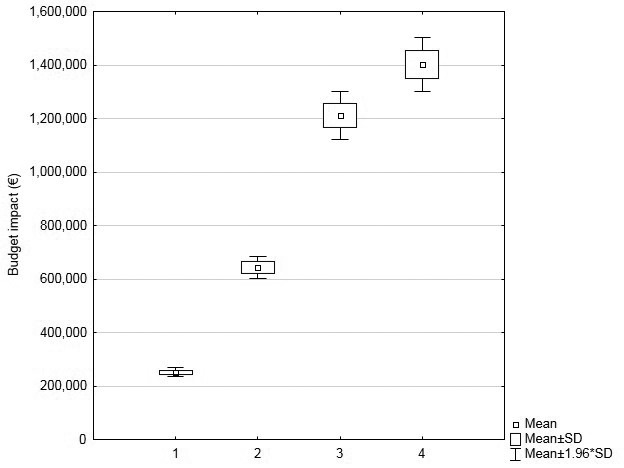
Impact on CHIF’s budget following different scenarios of OBV/PTV/r/DSV entering the market. Scenarios: 1 – OBV/PTV/r/DSV takes over only the market shares of rival therapies that have higher costs per SVR; 2 – OBV/PTV/r/DSV additionally takes over the patient subgroups for which its average costs per SVR are more favorable, but the range of modeled savings per SVR is such that there is also a chance for OBV/PTV/r/DSV to be more expensive than the rival therapies; 3 – additionally to two previous scenarios, OBV/PTV/r/DSV takes over the market shares of the rival therapies that have up to €10 000 lower average costs per SVR; 4 – OBV/PTV/r/DSV takes over the entire market. CHIF – Croatian Health Insurance Fund, OBV/PTV/r/DSV – ombitasvir, paritaprevir, ritonavir, and dasabuvir, SVR – sustained virological response.

Since the cost-effectiveness analysis showed that for naive patients the standard dual pegIFN therapy was the cheapest one compared to alternative regimens regardless of cirrhosis status (Figure 1), we assumed it remains the standard of care (SOC) for naive HCV GT1 population, and this group of patients was not included in BIA. The BIA was performed in order to calculate the financial impact of market inclusion of OBV/PTV/R/DSV therapy on CHIF’s budget. To better capture the incremental effect of the changes, four BIA scenarios that systematically increased the OBV/PTV/r/DSV market shares according to increases in OBV/PTV/r/DSV costs per achieved SVR (Figures 3A and 3B) were developed. In the first scenario, the OBV/PTV/r/DSV therapy took over only the market shares of the competitors that were less cost-effective per SVR under simulated circumstances. In the second scenario, the OBV/PTV/r/DSV therapy took over the market shares of HCV GT1 experienced patients for whom OBV/PTV/r/DSV average costs per SVR were lower than those of the alternative regimens, but there still existed a possibility that the costs could be greater as well. The third scenario implied that OBV/PTV/r/DSV took over the market shares for which it was on average €10 000 more expensive in comparison to the rivals. In the final scenario, OBV/PTV/r/DSV took over the entire market. According to described scenarios, OBV/PTV/r/DSV market entry would cost CHIF additional €0.25-1.4 mil per year (Figure 3), which represents expenditure increase of 8%, 20%, 37%, and 43% for respective scenarios.

### Increase in the number of successfully treated patients and expenditures per achieved SVR

**Figure 4:**
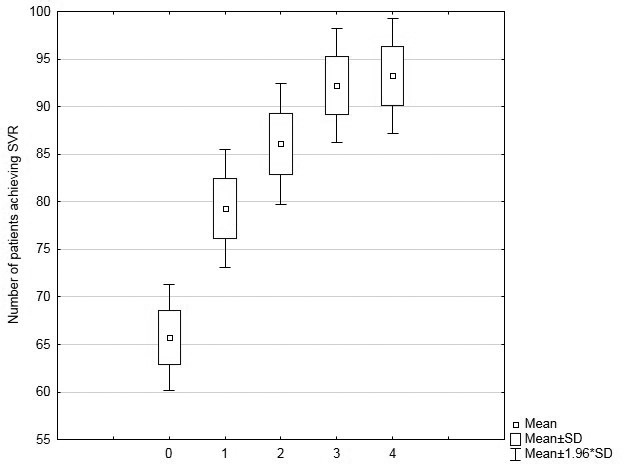
The number of successfully treated patients according to the following scenarios: 0 – only rival therapies present on the market; 1 – OBV/PTV/r/DSV takes over only the market shares of rival therapies that have higher costs per SVR; 2 – OBV/PTV/r/DSV additionally takes over the patient subgroups for which its average costs per SVR are more favorable, but the range of modeled savings is such that there is also a chance for OBV/PTV/r/DSV to be more expensive than the rival therapies; 3 – additionally to two previous scenarios, OBV/PTV/r/DSV takes over the market shares of the rival therapies that have up to €10 000 lower average costs per SVR; 4 – OBV/PTV/r/DSV takes over the entire market. OBV/PTV/r/DSV – ombitasvir, paritaprevir, ritonavir, and dasabuvir, SVR – sustained virological response.

**Figure 5:**
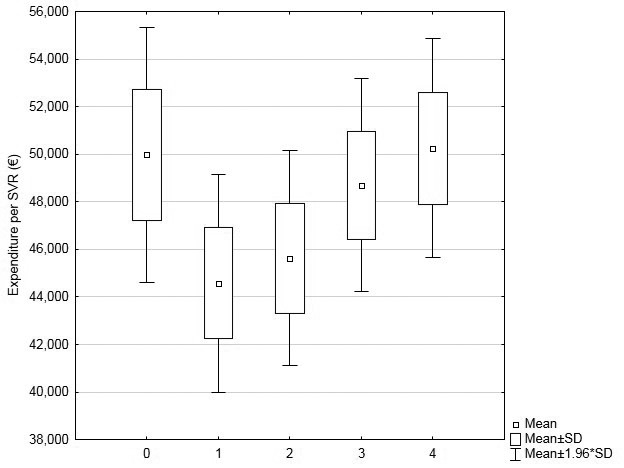
Expenditure per SVR according to the following scenarios: 0 – only rival therapies present on the market; 1 – OBV/PTV/r/DSV takes over only the market shares of rival therapies that have higher costs per SVR; 2 – OBV/PTV/r/DSV additionally takes over the patient subgroups for which its average costs per SVR are more favorable, but the range of the savings is such that there is also a chance for OBV/PTV/r/DSV to be more expensive than the rival therapies; 3 – additionally to two previous scenarios, OBV/PTV/r/DSV takes over the market shares of the rival therapies that have up to €10 000 lower average costs per SVR; 4 – OBV/PTV/r/DSV takes over the entire market. OBV/PTV/r/DSV – ombitasvir, paritaprevir, ritonavir, and dasabuvir, SVR – sustained virological response.

In order to compare the performance of the OBV/PTV/r/DSV and alternative regimens, Monte Carlo simulations were carried out to calculate the percentage of experienced patients who would achieve SVR depending on OBV/PTV/r/DSV market shares. Simulations were performed according to the same scenarios used in BIA. The number of successfully treated patients significantly increased with every consecutive scenario (*P* < 0.001) (Figure 4). Without OBV/PTV/r/DSV on the market, the average percentage of patients achieving SVR was 69%, while with OBV/PTV/r/DSV on the market this percentage ranged from 83% (for the scenario where OBV/PTV/r/DSV takes over only the market shares for which it would be the cheapest therapy per SVR) up to 98% (for the scenario where OBV/PTV/r/DSV takes over the entire market). This means that the introduction of OBV/PTV/r/DSV would, in analyzed scenarios, on average increase the number of successfully treated patients by 20%, 30%, 40%, and 42%, respectively (Figure 4). Considering expenditures per achieved SVR, OBV/PTV/r/DSV was proven to be cost-effective for the first three scenarios (*P* < 0.001), while in the fourth scenario costs per SVR were slightly higher than in the scenario without OBV/PTV/r/DSV (*P* < 0.001) (Figure 5).

### Incremental cost-effectiveness ratio

**Figure 6:**
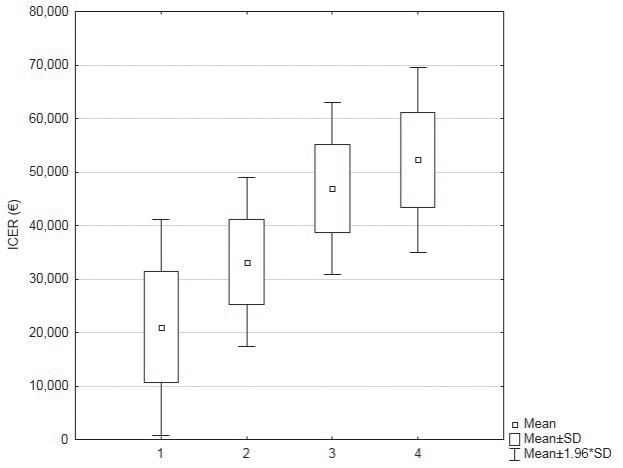
Additional expenditure per additional SVR according to the following scenarios: 1 – OBV/PTV/r/DSV takes over only the market shares of rival therapies that have higher costs per SVR; 2 – OBV/PTV/r/DSV additionally takes over the patient subgroups for which its average costs per SVR are more favorable, but the range of the savings is such that there is also a chance for OBV/PTV/r/DSV to be more expensive than the rival therapies; 3 – additionally to two previous scenarios, OBV/PTV/r/DSV takes over market shares of the rival therapies that have up to €10 000 lower average costs per SVR; 4 – OBV/PTV/r/DSV takes over the entire market. OBV/PTV/r/DSV – ombitasvir, paritaprevir, ritonavir, and dasabuvir, SVR – sustained virological response.

ICER represents additional cost per SVR from an additional market share of OBV/PTV/r/DSV taken over from the competition. The average ICER from the lowest OBV/PTV/r/DSV market expansion was approximately €21,000, and in the scenario when OBV/PTV/r/DSV takes over the entire market of HCV GT1 experienced population, the average ICER reaches €52 000 (Figure 6).

## Discussion

By using the marginal cost-effectiveness approach to decide on the order of introduction of new therapies as inputs into BIA, we showed that, in spite of its high price, the new combination therapy could still be acceptable even for a less wealthy EU MS. As an example we used OBV/PTV/r/DSV therapy for HCV GT1 treatment. OBV/PTV/r/DSV protocols take half the time the first generation PI therapies take, and clinical trials indicate their high efficacy. Since shorter treatments with fewer side-effects increase adherence to treatments (27), higher effectiveness of OBV/PTV/r/DSV compared to the first generation PI therapies may also be expected.

In the case of OBV/PTV/r/DSV combination therapy, the mentioned approach resulted in four scenarios based on discrete market shares provided by each scenario. According to these scenarios, an average rise in expenditure of 8%, 20%, 37%, and 43%, would result in an average increase of 20%, 30%, 40%, and 42% of successfully treated patients, respectively. In the first scenario, the OBV/PTV/r/DSV therapy takes over the market shares of less cost-effective competitors, ie, a large portion of patients with cirrhosis and all null responders without cirrhosis. So, in the cases where cirrhotic patients were the dominant group of experienced HCV population, the relation of an expenditure increase and an increase in the number of successfully treated patients may be even more favorable.

Therefore, we can say that the introduction of OBV/PTV/r/DSV combination therapy onto the CHIF reimbursement list would have a significant budget impact, but there also might be expected a substantial increase in the potential number of cured persons. We found the results of the simulation of the expenditure per SVR especially interesting, since they showed the costs per potentially cured person to be actually lower for the OBV/PTV/r/DSV combination therapy than for rival therapies in all examined scenarios but the last one, which is the least possible as it assumes complete market takeover by OBV/PTV/r/DSV.

The findings of the analysis are in accordance with the latest Croatian recommendations for the treatment of chronic hepatitis C GT1, where OBV/PTV/r/DSV has been recommended for treating all cirrhotic patients and null-responders (Table 3) (7). In the recommendations, pegIFN remained SOC for naive patients with fibrosis stage F1-F2, and the first generations PI were replaced by OBV/PTV/r/DSV, SIM-, and SOF-based therapies. It is important to mention that SOF was not included in the study, since SOF and OBV/PTV/r/DSV entered the Croatian market simultaneously.

**Table 3:** Croatian recommendations for the treatment of HCV GT1 (7)*

HCV GT1 patient subgroup	Fibrosis score	Therapy
Naive	F1-F2	pegIFN
	F3	SIM+pegIFN, SOF+pegIFN
	F4/CC	OBV/PTV/r/DSV, SOF+SIM
Relapsers and partial responders	F1-F3	SIM+pegIFN, SOF+pegIFN
	F4/CC	OBV/PTV/r/DSV, SOF+SIM
Null responders	F1-F4/CC	OBV/PTV/r/DSV, SOF+SIM

*HCV GT1 – hepatitis C virus genotype 1; pegIFN – pegylated interferon; OBV/PTV/r/DSV – ombitasvir, paritaprevir, ritonavir and dasabuvir; SIM – simeprevir; SOF – sofosbuvir; CC – compensated cirrhosis.

Limitations of our study were a lack of precise epidemiological data, as well as the fact that study included only direct therapies’ costs. Presented results were obtained exclusively by quantitative analysis, and their interpretation was dominantly economic, while the final decision on the medication’s inclusion onto a reimbursement list requires interdisciplinary work, including detailed medical/pharmacological considerations.

We showed how highly effective but extremely expensive drugs may be acceptable for middle and low income EU countries. Nevertheless, as a consequence of EU regulation and the inappropriateness of price discrimination, we expect an increase in confidential agreements between pharmaceutical industry and health insurance companies, with quantity based rebates, and pay-per-cure or other similar risk-sharing arrangements. Therefore, there is a necessity for negotiations between national health insurance funds and the pharmaceutical industry, not only concerning this particular case, but also concerning every other expensive therapy. These negotiations may lead to differential prices at international level that might seem unfair, but are both allocatively efficient and welfare increasing for a small and low-income country (28). We propose to alleviate this problem by basing all health-economic outcomes of new drugs/therapies on their cost-effectiveness analyses. Cost-effectiveness analysis provides the introduction dynamics of a new combination therapy for particular patient subgroups, which can serve as a basis for budget impact and other connected analyses.

Emergence of new therapies can be both thrilling because of remarkable efficacy and puzzling because of extremely high prices. The change in the treatment of HCV infection is probably one of the most current examples of such a situation. In order to include the newest therapies on reimbursement lists of middle and low income EU member states, we propose to put the cost-effectiveness of new drug therapies on the forefront of the analysis. The new therapy should first be introduced to groups of patients for which it has the lowest price per successfully treated person, followed by increasingly more expensive scenarios per successful treatment. The analysis was performed from the economic point of view, but the final decision on drug inclusion onto the reimbursement list requires interdisciplinary work.
